# Misophonia in Individuals with Obsessive–Compulsive Disorder: Its Relationship with Anxiety Sensitivity and Mindfulness

**DOI:** 10.3390/medicina62010216

**Published:** 2026-01-20

**Authors:** Mahmut Onur Karaytuğ, Lut Tamam, Mehmet Emin Demirkol, Zeynep Namlı, Caner Yeşiloğlu, Sinem Çetin Demirtaş, Ali Meriç Kurt, Hale Nur Çakar, Efsun Damla Altın, Mahmut Gürbüz

**Affiliations:** 1 Faculty of Medicine, Çukurova University, Sarıçam 01330, Türkiye; 2 Nizip State Hospital, Gaziantep 27700, Türkiye; 3 St. Elisabeth Krankenhaus, 45529 Hattingen, Germany

**Keywords:** obsessive–compulsive disorder, misophonia, anxiety sensitivity, mindfulness, mediation analysis

## Abstract

*Background and Objectives*: This study aimed to examine the severity of misophonia symptoms in individuals diagnosed with obsessive–compulsive disorder (OCD) and to evaluate the pattern of the relationship between misophonia and OCD symptom severity in relation to anxiety sensitivity and mindfulness. *Materials and Methods*: This comparative and cross-sectional study included 108 patients diagnosed with OCD according to DSM-5 criteria and 81 healthy control subjects without any psychiatric diagnosis. Participants completed the Misophonia Symptom List (MSL), Anxiety Sensitivity Index-3 (ASI-3), Mindful Attention Awareness Scale (MAAS), Yale–Brown Obsessive Compulsive Scale (Y-BOCS), and Beck Anxiety Inventory (BAI). Statistical analyses included group comparisons, Pearson correlations, multiple linear regression, and mediation analyses using the PROCESS macro. *Results*: MSL scores were significantly higher in the OCD group compared to the control group (104.10 ± 33.00 vs. 87.56 ± 20.07, *p* < 0.001). ASI-3 (33.53 ± 18.72 vs. 18.12 ± 11.55, *p* < 0.001) and BAI scores (20.74 ± 13.14 vs. 11.04 ± 8.47, *p* < 0.001) were higher; MAAS scores were lower (53.23 ± 14.92 vs. 60.72 ± 12.70, *p* < 0.001). In the OCD group, MSL scores were positively correlated with anxiety sensitivity (r = 0.626, *p* < 0.001) and Beck anxiety (r = 0.515, *p* < 0.001) and negatively correlated with MAAS (r = −0.357, *p* < 0.001). In multiple regression analysis, anxiety sensitivity was identified as the only variable significantly predicting misophonia severity (β = 0.523, *p* < 0.001). Mediation analyses showed that anxiety sensitivity emerged as the dominant indirect pathway between OCD symptom severity and misophonia, whereas the contribution of mindfulness was not independent of anxiety sensitivity in the serial mediation model. *Conclusions*: The findings indicate that misophonia symptoms are significantly elevated in individuals diagnosed with OCD and that these symptoms are particularly associated with cognitive-emotional variables such as anxiety sensitivity and mindfulness. Given the cross-sectional design, the mediation findings should be interpreted as indirect associations rather than evidence of causal pathways. Considering these variables may be useful in assessing misophonia symptoms accompanying OCD and planning clinical approaches.

## 1. Introduction

Obsessive–Compulsive Disorder (OCD) is a chronic mental disorder characterized by obsessions—intrusive and distressing thoughts—and compulsions—repetitive behaviors or mental acts performed to reduce distress. Its lifetime prevalence is approximately 2–3%, with onset typically occurring during adolescence or early adulthood, often leading to marked functional impairment [1]. Etiological models of OCD emphasize the interaction of genetic vulnerability, neurobiological mechanisms, maladaptive cognitive processes, and learned behaviors. In addition to core obsessive-compulsive symptoms, impairments in higher-order cognitive-emotional processes such as emotion regulation and impulse control are frequently observed and are increasingly recognized as central to symptom maintenance [2].

Misophonia, derived from the Greek words *misos* (hatred) and *phonia* (sound), refers to a decreased tolerance to specific sounds and was first introduced into the scientific literature by Jastreboff and Jastreboff in the early 2000s. It is classified among “low sound tolerance” conditions alongside hyperacusis and phonophobia. Individuals with misophonia experience intense emotional and physiological reactions—most commonly anger, disgust, and anxiety—in response to specific auditory triggers, which are predominantly human-generated sounds but may also include mechanical or animal sounds [3,4]. These responses are often accompanied by marked difficulties in emotion regulation. Accumulating evidence suggests conceptual and phenomenological overlap between misophonia and OCD. Both conditions involve heightened threat perception, difficulty disengaging from aversive internal or external stimuli, and repetitive regulatory behaviors aimed at reducing distress. On this basis, some authors have proposed that misophonia may share pathophysiological mechanisms with OCD or even fall within the obsessive-compulsive spectrum [5,6]. Empirical findings support this view, with Jager et al. reporting that 2.8% of individuals diagnosed with misophonia also met diagnostic criteria for OCD [7]. However, the cognitive-emotional mechanisms underlying this association remain insufficiently understood.

Anxiety sensitivity (AS)—the tendency to interpret anxiety-related physical, cognitive, or social sensations as harmful—has been identified as a transdiagnostic vulnerability factor across anxiety-related disorders [8]. Individuals with OCD exhibit some of the highest AS levels outside of panic disorder, and AS has been shown to predict symptom severity in OCD and related conditions [9,10]. Cognitive dimensions of AS, particularly fear of losing cognitive control, appear closely linked to obsessive thought patterns and maladaptive attempts at thought suppression. Moreover, AS has been positively associated with misophonia severity, suggesting that heightened sensitivity to internal anxiety cues may amplify emotional reactivity to auditory triggers.

Mindfulness, defined as deliberate and non-judgmental awareness of present-moment experience, represents another cognitive-emotional process that may be relevant to both OCD and misophonia [11]. According to Bishop et al.’s two-component model, mindfulness involves both attentional regulation and an accepting orientation toward experience [12]. Lower mindfulness levels have been associated with increased psychopathology, whereas mindfulness-based interventions have demonstrated efficacy in reducing OCD symptoms and improving quality of life [13,14]. Although research on mindfulness in misophonia is limited, emerging evidence suggests that mindfulness-based approaches may reduce symptom severity by enhancing emotional regulation and decreasing reactive responses to auditory triggers [15,16].

It has been reported that the intense emotional responses accompanying misophonia are not limited to sensory sensitivity alone; triggering stimuli elicit strong emotional responses in individuals associated with increased internal arousal and threat perception [17,18,19]. In this context, anxiety sensitivity represents the emotional reactivation dimension of misophonia, while mindfulness reflects the capacity to regulate these responses at the cognitive level. Considering these two constructs within the same model, along with OCD symptom severity, allows us to distinguish whether misophonia is primarily shaped by emotional vulnerability or cognitive regulation processes in the context of OCD.

Accordingly, the present study aims to examine the relationship between OCD symptom severity and misophonia symptoms and to test whether anxiety sensitivity and mindfulness play explanatory roles in this association. It is hypothesized that higher OCD symptom severity will be associated with greater misophonia symptoms, that anxiety sensitivity will be positively associated with both OCD and misophonia, and that mindfulness will be negatively associated with symptom severity. Furthermore, it is hypothesized that anxiety sensitivity and mindfulness will mediate the relationship between OCD symptom severity and misophonia symptoms.

## 2. Method

The study sample consisted of individuals aged 18–65 who were diagnosed with OCD according to DSM-5 diagnostic criteria and were recruited from the Obsessive–Compulsive Disorder outpatient clinic of the Psychiatry Department at Çukurova University Faculty of Medicine between August 2025 and October 2025. All patients presenting to the OCD outpatient clinic during this period were initially screened for eligibility (n = 133). Individuals with psychotic disorders, bipolar disorder, alcohol or substance use disorders, intellectual disability, or illiteracy were excluded from the OCD group. Of the remaining patients, 15 declined participation, 7 met criteria for a current major depressive disorder, and 3 were excluded due to mild cognitive impairment. Following these exclusions, the final OCD sample consisted of 108 participants. All clinical assessments were conducted face-to-face by a psychiatrist. The diagnosis of OCD was confirmed using a DSM-5–based clinical interview, and potential psychiatric comorbidities were systematically assessed and excluded in accordance with the predefined exclusion criteria. In addition, participants with a current diagnosis of major depressive disorder were excluded in order to minimize the potential confounding effects of depressive symptomatology on anxiety sensitivity, mindfulness, and misophonia-related symptoms. The control group was recruited from hospital staff and their relatives during the same time period. Control participants were interviewed face-to-face, and psychiatric history was obtained through a DSM-5–based clinical interview conducted by a psychiatrist. The absence of any psychiatric diagnosis according to DSM-5, as well as no history of psychiatric treatment or medication use, were set as the main inclusion criteria for the control group. The OCD and control groups were matched at the group level in terms of age, gender, and years of education to ensure comparability between samples. In both groups, individuals with anatomical abnormalities in the external ear canal that could cause hearing impairment, those who reported hearing loss, or those who used hearing aids were excluded from the study. The sample size was calculated using the G*Power 3.1 program for multiple linear regression analysis with a medium effect size (f^2^ = 0.15), α = 0.05, 95% power (1 − β = 0.95), and four predictor variables; it was determined that at least 129 participants were required. The study was approved by the Çukurova University Faculty of Medicine Non-Interventional Clinical Research Ethics Committee on 18 July 2025, with decision number 55.

### 2.1. Data Collection Tools

#### 2.1.1. Sociodemographic Data Form

The Sociodemographic Data Form, created by the researchers, consists of questions assessing variables such as age, gender, years of education, marital status, occupation, and place of residence.

#### 2.1.2. Misophonia Symptom List (MSL)

The Misophonia Symptom List (MSL) was used in this study to assess misophonia symptoms. The MSL is a form that inquires about the level of discomfort toward 50 different sound stimuli and enables the quantitative assessment of misophonia severity. The MSL uses a four-point Likert-type scale (1 = not at all, 2 = a little, 3 = moderately, 4 = very much), with total scores ranging from 50 to 200. A higher total score indicates more severe misophonia symptoms. In this study, only the symptom severity subscale of the MSL, which quantitatively measures the severity of discomfort toward triggering sounds, was used. This choice was made to reduce the response burden associated with applying multiple scales in the OCD sample and to focus on the component that directly measures ‘misophonia symptom severity,’ the primary outcome of the study.

#### 2.1.3. Mindful Attention Awareness Scale (MAAS)

Developed by Brown and Ryan, the Mindful Attention Awareness Scale (MAAS) is a single-factor construct that measures individuals’ tendencies to stay in the present moment and notice their experiences without judgment [20]. The scale was adapted into Turkish in 2011 by Özyesil, Arslan, Kesici, and Deniz. The scale is administered on a 6-point Likert scale. In Turkish psychometric analyses, the Cronbach’s alpha internal consistency coefficient was reported as 0.80, and the test–retest correlation was 0.86. These values indicate that the Turkish version of the MAAS is an internally consistent and stable measurement tool [21].

#### 2.1.4. Anxiety Sensitivity Index-3 (ASI-3)

To assess anxiety sensitivity, the Anxiety Sensitivity Index-3 (ASI-3) developed by Taylor and colleagues [22] and adapted into Turkish by Mantar, Yemez, and Alkın in 2010 was used [8]. The scale consists of 18 items organized into three subscales: Physical, Social, and Cognitive, and is answered on a five-point Likert scale. No cutoff score was determined for the Turkish adaptation, and high scores indicate increased anxiety sensitivity. In the validity and reliability study, Cronbach’s alpha values were found to be 0.89, 0.82, and 0.88 for the physical, social, and cognitive subscales, respectively, and 0.93 for the entire scale.

#### 2.1.5. Yale–Brown Obsession and Compulsion Scale (Y-BOCS)

Developed by Goodman and colleagues to assess the types and severity of obsessions and compulsions, the Yale–Brown Obsessive Compulsive Scale (Y-BOCS) consists of 19 items [23]. The Turkish validity and reliability study was conducted by Karamustafalıoğlu and colleagues, and the Cronbach’s alpha coefficient was reported as 0.81 [24]. The total score ranges from 0 to 40, and when scoring, items 1b and 6b are excluded, and the first 10 items are evaluated; the first five items measure obsession, and the next five items measure compulsion severity. The other items in the scale are not included in the total score.

#### 2.1.6. Beck Anxiety Inventory (BAI)

Developed by Beck and colleagues in 1988, the Beck Anxiety Inventory (BAI) is a 21-item self-report instrument that measures the severity of anxiety-specific somatic and cognitive symptoms [25]. Each item is scored on a scale of 0–3, with the total score ranging from 0 to 63. The Turkish validity and reliability study of the scale was conducted by Ulusoy and colleagues in 1998, and the Cronbach’s alpha coefficient was reported as 0.93 [26].

### 2.2. Statistics

Statistical analyses were performed using IBM SPSS Statistics for Windows, Version 26.0 (IBM Corp., Armonk, NY, USA). A significance level of *p* < 0.05 was accepted for all analyses. The distribution characteristics of continuous variables were examined using descriptive statistics (mean and standard deviation), and the variables were evaluated to determine whether they met the assumptions of parametric tests.

Statistical analyses were conducted in accordance with the predefined study hypotheses. Primary analyses included between-group comparisons and mediation models designed to examine the associations among OCD symptom severity, misophonia symptom severity, anxiety sensitivity, and mindfulness. Specifically, group differences in misophonia symptoms, anxiety sensitivity, and mindfulness were examined between the OCD and control groups. In addition, correlational analyses were performed to assess the associations between OCD symptom severity, anxiety sensitivity, mindfulness, and misophonia symptom severity. To test the hypothesized explanatory roles of anxiety sensitivity and mindfulness, mediation analyses were conducted to examine whether these variables accounted for indirect associations between OCD symptom severity and misophonia symptoms. These mediation models were specified a priori based on the study hypotheses. Given the cross-sectional design, mediation analyses were interpreted as statistical models of indirect association rather than evidence of causal pathways. Additional correlational analyses conducted beyond the primary hypotheses were considered exploratory and are reported to provide descriptive and hypothesis-generating information. Statistical significance was evaluated using two-tailed tests, and effect sizes and confidence intervals were reported to facilitate interpretation of the magnitude of associations.

For the comparison of sociodemographic variables between the OCD and control groups, the chi-square test was used for categorical variables and the independent samples *t*-test for continuous variables. The independent samples *t*-test was also applied in group comparisons of scale scores due to the fulfillment of parametric assumptions. Since the Y-BOCS was only administered to the OCD group, no group comparison was made for this scale.

The relationships between psychometric variables in the OCD group were evaluated using Pearson correlation analysis. Correlation coefficients (r) and corresponding significance levels were reported.

To examine the variables predicting misophonia symptoms, single linear regression analyses were first performed, and the independent effects of Y-BOCS, MAAS, ASI-3, and BAI variables on misophonia symptoms were evaluated separately. Subsequently, a multiple linear regression analysis was performed with all variables included in the same model. In the multiple regression model, multicollinearity was tested using the Variance Inflation Factor (VIF), and since the VIF values for all variables were <5, the absence of multicollinearity in the model was confirmed. A hierarchical multiple regression analysis was conducted to examine the incremental contribution of anxiety sensitivity and mindfulness to the prediction of misophonia symptom severity beyond OCD symptom severity. Y-BOCS scores were entered in the first step, followed by ASI-3 and MAAS scores in the subsequent step. Changes in explained variance (ΔR^2^) were evaluated to determine the added explanatory value of these variables.

To evaluate the potential mediating roles of anxiety sensitivity and mindfulness in the relationship between Y-BOCS and MSL, two separate mediation analyses were conducted using the PROCESS add-on (Hayes, Model 4, IBM Corp., Calgary, AB, Canada) [27]. In the models, ASI-3 and MAAS were defined as mediator variables, respectively, and the significance of indirect effects was tested using 5000 bootstrap samples with a 95% confidence interval. For each model, the standardized coefficients, standard errors, and *p*-values for paths a, b, c (total effect), and c′ (direct effect) were reported.

In addition, a serial multiple mediation analysis was conducted using the PROCESS macro (Hayes, Model 6, IBM Corp., Calgary, AB, Canada) [27] to examine the indirect association between OCD symptom severity and misophonia symptoms through anxiety sensitivity and mindfulness in sequence. In this model, Y-BOCS scores were specified as the independent variable, anxiety sensitivity and mindfulness were entered as serial mediators, and misophonia symptom severity was defined as the outcome variable. Indirect effects were tested using 5000 bootstrap samples with 95% confidence intervals. Consistent with the cross-sectional design, this analysis was interpreted as a statistical model of indirect association rather than evidence of causal pathways.

## 3. Results

Table 1 presents the sociodemographic characteristics of the OCD and control groups. There was no significant difference between the groups in terms of age (*p* = 0.268). No significant difference was found between the groups in terms of years of education (*p* = 0.207). No significant differences were observed between the two groups in terms of gender distribution (*p* = 0.966), marital status (*p* = 0.267), occupational status (*p* = 0.078), and place of residence (*p* = 0.637).

In the present sample, the internal consistency of the study measures ranged from good to excellent. MSL symptom severity score demonstrated excellent internal consistency (Cronbach’s α = 0.84), ASI-3 showed excellent internal consistency (Cronbach’s α = 0.85), and MAAS demonstrated good internal consistency (Cronbach’s α = 0.88). The BAI showed excellent internal consistency (Cronbach’s α = 0.80), and the Y-BOCS demonstrated good internal consistency (Cronbach’s α = 0.82).

Table 2 compares the scale scores of the OCD and control groups. The OCD group’s MSL, ASI-3, and BAI scores were found to be significantly higher than the control group *p* < 0.001, Cohen’s d = 0.59; *p* < 0.001, Cohen’s d = 0.96; *p* < 0.001, Cohen’s d = 0.85), respectively). The MAAS score was found to be significantly higher in the control group (*p* < 0.001, Cohen’s d = 0.53).

Table 3 shows the correlations between scale scores in the OCD group. The Y-BOCS score was negatively correlated with MAAS scores (r = −0.309, *p* = 0.001) and positively correlated with MSL (r = 0.319, *p* = 0.001), and ASI-3 scores (r = 0.486, *p* < 0.001). The MAAS scores were found to be negatively correlated with MSL (r = −0.357, *p* < 0.001), ASI-3 (r = −0.549, *p* < 0.001) and BAI scores (r = −0.572, *p* < 0.001). The MSL score was positively correlated with ASI-3 (r = 0.626, *p* < 0.001) and BAI scores (r = 0.515, *p* < 0.001). A positive correlation was observed between ASI-3 and BAI scores (r = 0.652, *p* < 0.001).

Table 4 presents the results of the multiple regression analysis in which Y-BOCS, MAAS, ASI-3, and BAI scores were included in the model together. The model was found to be significant overall (F = 18.118, *p* < 0.001). When the variables were evaluated together, ASI-3 was found to predict MSL scores at a significant level (β = 0.523, *p* < 0.001). The BAI score showed a trend close to a significant contribution level to the model (β = 0.206, *p* = 0.059). The Y-BOCS score (β = −0.015, *p* = 0.861) and the MAAS score (β = 0.043, *p* = 0.651) did not show a significant contribution to the model. The VIF values of all variables were found to be below 5, and it was determined that there was no multicollinearity problem in the model. In the multiple model, only anxiety sensitivity remained as an independent variable that significantly predicted misophonia symptoms.

In Table 5, to clarify the incremental contribution of anxiety sensitivity, a hierarchical regression analysis was conducted in the OCD group. In the first step, Y-BOCS, BAI, and MAAS scores were entered as covariates, explaining 21.8% of the variance in misophonia symptom severity (R^2^ = 0.218). In the second step, resulting in a significant increase in explained variance when analysis with ASI (ΔR^2^ = 0.211, F(1103) = 38.18, *p* < 0.001).

**Figure 1:**
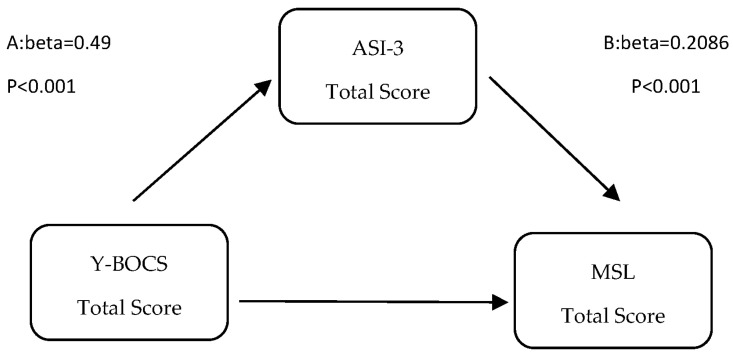
The mediating role of ASI-3 in the relationship between Y-BOCS and MSL.

The mediation model examining the indirect association between obsessive–compulsive symptom severity and misophonia symptom severity through anxiety sensitivity is illustrated in Figure 1. Table 6a presents the results of the mediation analysis examining the relationships among Y-BOCS, ASI-3, and MSL. The Y-BOCS score was significantly related to ASI-3 (path a: β = 0.49, SE = 0.085, *p* < 0.001). ASI-3, in turn, was significantly related to MSL scores (path b: β = 0.62, SE = 0.087, *p* < 0.001). The overall relationship between Y-BOCS and MSL was statistically significant (path c: β = 0.32, SE = 0.092, *p* = 0.001). However, when ASI-3 was taken into account, the direct pathway between Y-BOCS and MSL was no longer statistically significant (path c′: β = 0.02, SE = 0.087, *p* = 0.82). Bootstrap analyses indicated that the indirect pathway through ASI-3 was statistically significant (ab = 0.30, SE = 0.063, *p* < 0.001). These findings indicate that ASI-3 plays a full mediating role in the relationship between Y-BOCS and MSL.

**Figure 2:**
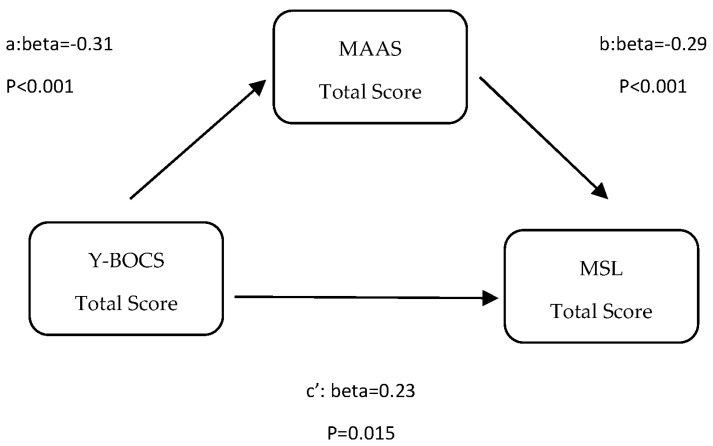
The mediating role of MAAS in the relationship between Y-BOCS and MSL.

The mediation model examining the indirect association between obsessive–compulsive symptom severity and misophonia symptom severity (MSL) through mindfulness is presented in Figure 2. Table 6b presents the results of the mediation analysis examining the relationships among Y-BOCS, MAAS, and MSL. The Y-BOCS score was significantly related to MAAS (path a: β = −0.31, SE = 0.092, *p* = 0.001). MAAS was, in turn, significantly related to MSL scores (path b: β = −0.29, SE = 0.093, *p* = 0.003). The overall relationship between Y-BOCS and MSL was statistically significant (path c: β = 0.32, SE = 0.092, *p* = 0.001). When MAAS was taken into account, the direct relationship between Y-BOCS and MSL remained statistically significant (path c′: β = 0.23, SE = 0.093, *p* = 0.015). Bootstrap analyses indicated that the indirect pathway through MAAS was statistically significant (ab = 0.09, SE = 0.044, *p* = 0.009). These findings indicate that MAAS plays a partial mediating role in the relationship between Y-BOCS and MSL.

**Figure 3:**
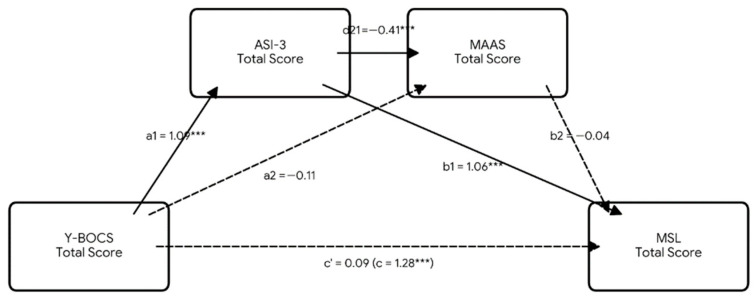
Serial mediation of Anxiety Sensitivity and Mindfulness in the relationship between Obsessive-Compulsive symptoms and Misophonia. Note: Y-BOCS: Yale–Brown Obsessive Compulsive Scale; ASI-3: Anxiety Sensitivity Index-3; MAAS: The Mindful Attention Awareness Scale; MSL: The Misophonia Symptom List. a1, a2, d21, b1, b2: path coefficients; c′: direct pathway. Solid lines indicate significant paths (*p* < 0.001), dashed lines indicate non-significant paths. ***: *p* < 0.001.

A serial multiple mediation model (PROCESS Model 6) [27] was used to describe the pattern of statistical pathways among obsessive–compulsive symptoms (Y-BOCS), anxiety sensitivity, mindfulness, and misophonia severity (Figure 3).

The model accounted for 39% of the variance in misophonia severity (R^2^ = 0.39, *p* < 0.001). Y-BOCS was positively associated with anxiety sensitivity (b = 1.09, SE = 0.18, *p* < 0.001). Anxiety sensitivity was negatively associated with mindfulness (b = −0.41, SE = 0.08, *p* < 0.001) and positively associated with misophonia severity (b = 1.06, SE = 0.18, *p* < 0.001). The association between Y-BOCS and mindfulness was not statistically significant (b = −0.11, SE = 0.16, *p* = 0.49), and mindfulness was not significantly associated with misophonia severity in the serial multiple mediation framework (b = −0.04, SE = 0.21, *p* = 0.85).

The total association between Y-BOCS and misophonia severity was significant (b = 1.28, SE = 0.35, *p* < 0.001). In the serial multiple mediation framework, the direct association between Y-BOCS and misophonia severity was not statistically significant (b = 0.09, SE = 0.34, *p* = 0.78). Bootstrap analyses showed that the indirect pathway linking Y-BOCS and misophonia severity through anxiety sensitivity was statistically significant (b = 1.16, 95% CI [0.66, 1.76]), whereas the indirect pathway through mindfulness and the serial pathway through anxiety sensitivity followed by mindfulness were not statistically significant.

A serial multiple mediation model (PROCESS Model 6) was used to describe the pattern of statistical pathways among Y-BOCS, ASI-3, MAAS, and MSL (Figure 3). In addition to the serial mediation model, anxiety sensitivity and mindfulness were examined simultaneously to address concerns regarding shared variance, yielding a convergent pattern in which anxiety sensitivity emerged as the primary indirect pathway.

The model accounted for 39% of the variance in misophonia symptom severity (R^2^ = 0.39, *p* < 0.001). Y-BOCS was positively associated with anxiety sensitivity (b = 1.09, SE = 0.18, *p* < 0.001). ASI-3 was negatively associated with mindfulness (b = −0.41, SE = 0.08, *p* < 0.001) and positively associated with MSL (b = 1.06, SE = 0.18, *p* < 0.001). The association between Y-BOCS and MAAS was not statistically significant (b = −0.11, SE = 0.16, *p* = 0.49), and MAAS was not significantly associated with MSL in the serial multiple mediation framework (b = −0.04, SE = 0.21, *p* = 0.85).

The total association between Y-BOCS and MSL was significant (b = 1.28, SE = 0.35, *p* < 0.001). In the serial multiple mediation framework, the direct association between Y-BOCS and MSL was not statistically significant (b = 0.09, SE = 0.34, *p* = 0.78).

Bootstrap analyses showed that the indirect pathway linking Y-BOCS and MSL through ASI-3 was statistically significant (b = 1.16, 95% CI [0.66, 1.76]), whereas the indirect pathway through mindfulness and the serial pathway through anxiety sensitivity followed by mindfulness were not statistically significant.

## 4. Discussion

One of the most important findings of our study is that anxiety sensitivity showed a pattern consistent with a mediating role in the association between Y-BOCS scores and misophonia symptom severity. The literature includes studies reporting that anxiety sensitivity is associated with misophonia [5] and that both anxiety sensitivity and OCD symptoms are significantly associated with misophonia cases [28]. It has been suggested that the threatening appraisal of intrusive thoughts in OCD is associated with heightened sensitivity to bodily sensations and increased levels of cognitive alarm, and that this pattern may be linked to elevated anxiety sensitivity [5,29]. The fact that misophonia is characterized by intense anger, disgust, and physiological arousal toward specific sounds suggests that these responses may be related to the increased internal arousal and threat perception seen in OCD [5,29]. Anxiety sensitivity can be considered a cognitive–emotional trait that may be related to misophonia symptoms, given its association with increased internal arousal and cognitive–emotional sensitivity accompanying OCD. Another important finding of our study is that mindfulness showed a pattern consistent with partial mediation in the relationship between Y-BOCS scores and misophonia symptom severity. Research on OCD reports lower levels of mindfulness and that this may increase emotional reactivity accompanying the threatening evaluation of intrusive thoughts [30]. Studies on misophonia have shown that mindfulness levels are associated with anger, disgust, irritability, and avoidance responses to triggering sounds, and that these responses can influence how they are evaluated [15,16]. Mindfulness-based approaches have been reported to reduce automatic responses to intrusive thoughts in OCD [30]; Similarly, mindfulness and acceptance-based practices for misophonia have been shown to alleviate emotional responses to triggering stimuli and avoidance behaviors [15,16]. When findings indicating that mindfulness is associated with both OCD symptoms and misophonic responses are considered together, differences in mindfulness levels may be related to the relationship between OCD and misophonia. However, because the data are cross-sectional, the observed mediation patterns do not establish temporal precedence or directionality and should be interpreted as indirect associations consistent with, but not demonstrating, causal pathways.

Our results demonstrated that the relationship between OCD symptoms and misophonia occurs primarily through increased anxiety sensitivity and that mindful awareness does not mediate this relationship. These results are consistent with studies showing that anxiety sensitivity is associated with threat-focused cognitive patterns and physiological sensitivity in OCD [29,31]. Andermane et al. reported that more intense reactions to triggering sounds are observed in individuals with high anxiety sensitivity [28]. Similarly, Schadegg et al. demonstrated that anxiety sensitivity is a process that intensifies the anger, hostility, and verbal aggression responses observed in misophonia [32]. The partial mediation finding of mindfulness observed in simple mediation models was not replicated in the serial mediation model. Although previous studies have reported that mindfulness-based interventions reduce both OCD symptoms and misophonic responses [15,16], the present findings suggest that the effect of mindfulness is not independent of anxiety sensitivity and that these two constructs should be evaluated together.

Misophonia is a condition characterized by intense anger, disgust, and physiological arousal in response to certain sounds, with pronounced emotional reactivity. The literature reports significant positive correlations between anxiety sensitivity and misophonia and shows that individuals with high anxiety sensitivity may have more intense reactions to triggering sounds [5,28]. In our study, anxiety sensitivity was identified as the only significant variable predicting misophonia symptom levels. This finding suggests that responses such as anger, disgust, and physiological arousal accompanying trigger sounds may stem from the cognitive-emotional sensitivity reflected by anxiety sensitivity. In line with this view, repetitive or intrusive auditory experiences, such as stuck song phenomena, have been described as distressing and clinically relevant in individuals with obsessive–compulsive features, highlighting the broader role of auditory intrusions in OCD-related symptomatology [33]. Anxiety sensitivity has been shown to function as a process that intensifies the anger, hostility, and verbal aggression responses observed in misophonia, and it has been reported that triggering stimuli create a more intense sense of threat and arousal in individuals with high anxiety sensitivity [32]. Andermane and colleagues also demonstrated that anxiety sensitivity is significantly higher in individuals with high levels of misophonia [28]. Importantly, hierarchical regression analyses demonstrated that anxiety sensitivity explained a substantial proportion of additional variance in misophonia severity beyond OCD symptom severity, general anxiety, and mindfulness, supporting its unique and incremental contribution to misophonic responses. In our study, a moderate association between anxiety sensitivity and general anxiety levels was an expected finding; however, these two constructs represent conceptually distinct dimensions. Anxiety sensitivity reflects fear of anxiety-related bodily and cognitive sensations and interpretations regarding their potential consequences, whereas the Beck Anxiety Inventory assesses the severity of current anxiety symptoms. Therefore, including both variables in the same regression model allows for the examination of their relatively independent contributions to misophonia symptoms. Our findings indicate that anxiety sensitivity remains associated with misophonia symptoms even after controlling for general anxiety levels. When considered together, these findings suggest that anxiety sensitivity may be an important component in the formation of physiological and emotional responses accompanying misophonia. An additional finding that warrants consideration is that OCD symptom severity was not a significant predictor of misophonia symptom severity when anxiety sensitivity was included in the regression model. This pattern suggests that misophonia symptoms in individuals with OCD may not be directly linked to the severity of obsessions and compulsions themselves but may instead be more closely related to heightened anxiety sensitivity. In this context, misophonia may function as a comorbid symptom cluster associated with increased interoceptive and threat sensitivity rather than as a core feature of obsessive–compulsive pathology. Nevertheless, given the cross-sectional nature of the data, this interpretation should be considered provisional, and longitudinal studies are needed to clarify the directionality and underlying mechanisms of these associations.

Our study revealed various relationships between OCD symptoms and the variables evaluated. Studies have reported that misophonia symptoms are more prevalent in individuals diagnosed with OCD and anxiety disorders and that obsessive cognitive processes may accompany misophonia responses [5,34]. In our study, the positive correlation between Y-BOCS and misophonia scores is consistent with the literature and suggests a possible link to the cognitive-emotional sensitivity reported in OCD. The hypervigilance and increased internal tension levels frequently observed in OCD may lead to a decrease in perceptual thresholds for environmental stimuli, thereby increasing the intensity of misophonic responses to triggering sounds. Experimental findings further suggest that affective responses to auditory stimuli, including music, may interact with attentional and working memory processes, indicating a potential link between sound-related emotional activation and cognitive functioning [35]. Associations between psychopathological symptoms, music preferences, and verbal working memory have been reported, supporting the notion that sound-related preferences and sensitivities may be embedded within broader cognitive and emotional processes [36]. Various studies have shown that mindfulness levels are lower in OCD and that threatening evaluations of intrusive thoughts reduce mindfulness. In our study, the negative relationship between Y-BOCS and mindfulness indicates that mindfulness capacity in OCD is associated with increased anxiety sensitivity due to cognitive loading and threat-focused evaluation processes, and that this sensitivity is related to symptom severity [29,31]. The positive relationship between anxiety sensitivity and Y-BOCS is explained by studies reporting that threat-sensitive cognitive patterns and physiological sensitivity accompanying intrusive thoughts in OCD can be seen alongside symptom increase [5,28,32,37]. Anxiety sensitivity may increase the intensity of emotional and physiological responses accompanying misophonia; it has been reported that anger, irritability, and distress responses to triggering sounds are more pronounced, especially in individuals with high sensitivity levels. The positive relationship between misophonia symptom severity and anxiety sensitivity in our study is consistent with these findings. Finally, it has been reported that low mindfulness levels are associated with anger, disgust, and avoidance responses to trigger sounds in misophonia, and that mindfulness-based interventions can reduce these responses [15,16]. The negative relationship between mindfulness and misophonia found in our findings is explained by these studies, which suggest that the emotional responses accompanying misophonia may be linked to attention processes and awareness capacity.

In our study, the finding that misophonia levels were higher in the OCD group compared to the control group is consistent with studies reporting that misophonia symptoms may be more severe in individuals diagnosed with OCD and anxiety disorders than in healthy controls [34,38]. The significantly higher anxiety sensitivity scores in the OCD group are consistent with studies reporting that increased internal arousal and threat-sensitive cognitive patterns in OCD are associated with anxiety sensitivity, whereas these processes are observed at lower levels in healthy individuals [29,31]. Previous research has shown that music preferences are associated with mental health characteristics across different populations, suggesting that affective responses to auditory stimuli may reflect broader cognitive–emotional patterns rather than isolated sensory reactions [39]. Consistent with this perspective, a systematic review has indicated that music-related phenomena, including musical obsessions and sound-related experiences, have been repeatedly described in individuals with OCD, supporting the relevance of auditory experiences within obsessive–compulsive symptom profiles [40]. The higher mindfulness levels found in the control group are consistent with the literature showing that mindfulness is inversely related to psychological processes such as rumination, avoidance, and emotional reactivity, and that stress responses may be lower in individuals with higher mindfulness levels.

The strengths of our study are that the relationships between anxiety sensitivity, mindfulness, and misophonia in individuals diagnosed with OCD were examined in the same sample. The consideration of these variables within a single model is not found in the literature. The use of group comparisons, correlation analyses, multivariate regression, and mediation models in the study allowed the findings to be tested using different methods. Determining the sample size based on power analysis and confirming clinical diagnoses through face-to-face interviews constitute another methodological strength. Considering the scarcity of research on misophonia in Turkey, our study contributes to the literature by presenting new data on sensory-emotional processes accompanying OCD. The findings of the study should be evaluated within the framework of some important methodological limitations. Because the study has a cross-sectional design, the relationships between variables cannot be interpreted causally. Although mediation analyses reveal statistical relationships, in this design, which does not include a time sequence, mediating relationships should be considered conceptually limited. Therefore, the mediation findings obtained should be evaluated as relational patterns between variables rather than causal mechanisms. Second, misophonia symptoms were assessed using a self-report measurement tool, and a structured clinical diagnostic interview was not administered. Furthermore, the use of only the symptom severity section of the scale limited the comprehensive assessment of the clinical dimensions and functional effects of misophonia. Third, although the psychiatric evaluation of the control group was conducted through a clinical interview based on DSM-5 diagnostic criteria, the absence of structured diagnostic interview instruments should be considered a limitation of the study, as the presence of possible subclinical symptoms cannot be entirely ruled out. In addition, information regarding illness duration and current pharmacological treatment was not systematically collected, which should be considered as a limitation when interpreting the findings. The fact that the variables included in the study were largely collected through self-report scales and in the same assessment session increases the possibility of common method variance. This should be considered as a factor that may affect the magnitude of the relationships between variables. Only anxiety sensitivity and mindful awareness were considered as variables in the study; other mechanisms that may be related to OCD and misophonia, such as emotion regulation, metacognitive processes, and behavioral avoidance, were not evaluated. Therefore, the findings reflect only a limited part of a broader cognitive-emotional model. The fact that only the section of the scale that quantitatively assesses symptom severity was used in the misophonia assessment should be considered a factor that may limit the generalizability of the findings to the contextual and phenomenological characteristics of misophonia.

## 5. Conclusions

Our study demonstrates that misophonia symptoms are significantly elevated in individuals diagnosed with OCD and that anxiety sensitivity and mindfulness levels play a crucial role in the underlying cognitive-emotional processes of this relationship. Anxiety sensitivity was not only associated with OCD symptoms but was also identified as a fully mediating mechanism in the relationship between OCD and misophonia symptoms. In addition, decreased mindfulness levels were found to be associated with the severity of misophonic responses and to play a partially mediating role in the OCD–misophonia symptom relationship. Taken together, the findings suggest that misophonia symptoms accompanying OCD are shaped not only by the phenomenology of the disorder but also by accompanying cognitive-emotional predispositions.

The findings suggest that incorporating anxiety sensitivity and mindfulness levels into routine clinical assessments may be useful in evaluating sensory-emotional responses accompanying OCD. Screening for misophonia symptoms in individuals diagnosed with OCD may allow for the consideration of responses to sensory triggers in clinical evaluation. The structuring of exposure applications in OCD treatment planning may be considered instructive in terms of evaluating sensory regulation approaches and addressing accompanying anxiety components. Considering that misophonia symptoms may be more intense in individuals with high anxiety sensitivity, integrating this component into treatment planning is important. Given that mindfulness-based interventions can reduce both the cognitive load of OCD symptoms and the emotional responses accompanying misophonia-related symptoms, adding these approaches as a supportive module to traditional OCD treatments may provide potential clinical benefits.

## Figures and Tables

**Table 1:** Sociodemographic Variables.

Variable	OCD (n = 108)	Control (n = 81)	*p*-Value	Effect Size Measures
**Age (mean ± SD)**	30.81 ± 10.75	32.77 ± 12.75	0.268	0.17 ^a^
**Gender, n (%)**			0.966	0.01 ^b^
Female (1)	61 (56.5%)	46 (56.8%)		
Male (2)	47 (43.5%)	35 (43.2%)		
**Education year (mean ± SD)**	14.30 ± 1.80	14.67 ± 2.10	0.207	0.19
**Marital status, n (%)**			0.267	0.06 ^b^
Single (1)	75 (69.4%)	50 (61.7%)		
Married (2)	33 (30.6%)	31 (38.3%)		
**Occupation, n (%)**			0.078	0.11 ^b^
Not working (1)	37 (34.3%)	38 (46.9%)		
Employed (2)	71 (65.7%)	43 (53.1%)		
**Place of residence, n (%)**			0.637	0.02 ^b^
County seat (1)	85 (78.7%)	66 (81.5%)		
Smaller than the provincial center (2)	23 (21.3%)	15 (18.5%)		

Data were analyzed using the chi-square test for categorical variables and the independent samples *t*-test for continuous variables. Coding: Gender (1 = Female, 2 = Male), Marital status (1 = Single, 2 = Married), Occupation (1 = Not working, 2 = Working), Place of residence (1 = County seat, 2 = Smaller than county seat). ^a^: Cohen’s d; ^b^: Cramer’s V.

**Table 2:** Comparison of Scale Scores Between the OCD and Control Groups.

Scale	OCD Group (n = 108) Mean ± SD	Control Group (n = 81) Mean ± SD	*p*-Value	Cohen’s d
**Yale-Brown Obsession and Compulsion Scale (Y-BOCS)**	19.06 ± 8.82	—	—	—
**Mindful Attention Awareness Scale (MAAS)**	53.23 ± 14.92	60.72 ± 12.70	<0.001	0.53
**Misophonia Symptom List (MSL)**	104.10 ± 33.00	87.56 ± 20.07	<0.001	0.59
**Anxiety Sensitivity Index-3 (ASI-3)**	33.53 ± 18.72	18.12 ± 11.55	<0.001	0.96
**Beck Anxiety** **Inventory (BAI)**	20.74 ± 13.14	11.04 ± 8.47	<0.001	0.85

Intergroup comparisons were performed using the independent samples *t*-test. Since the Y-BOCS was only administered to the OCD group and not evaluated in the control group, no statistical comparison was made for this scale. Values for other scales are reported as mean ± standard deviation.

**Table 3:** Correlations Between Scale Scores in the OCD Group.

Scales	Yale-Brown Obsession and Compulsion Scale (Y-BOCS)	Mindful Attention Awareness Scale (MAAS)	Misophonia Symptom List (MSL)	Anxiety Sensitivity Index-3 (ASI-3)	Beck Anxiety Inventory (BAI)
**Yale-Brown Obsession and Compulsion Scale (Y-BOCS)**	—	r = −0.309 *p* = 0.001	r = 0.319 *p* = 0.001	r = 0.486 *p* < 0.001	r = 0.455 *p* < 0.001
**Mindful Attention Awareness Scale (MAAS)**		—	r = −0.357 *p* < 0.001	r = −0.549 *p* < 0.001	r = −0.572 *p* < 0.001
**Misophonia Symptom List (MSL)**			—	r = 0.626 *p* < 0.001	r = 0.515 *p* < 0.001
**Anxiety Sensitivity Index-3 (ASI-3)**				—	r = 0.652 *p* < 0.001

Analyses were performed using the Pearson correlation method in the OCD group. The table reports correlation coefficients (r) and corresponding significance levels (*p*).

**Table 4:** Multivariate Regression Model Explaining Misophonia Symptoms.

Variable	B	β	SE	t	*p*	95% CI (Lower–Upper)	VIF
**Yale-Brown Obsession and Compulsion Scale (Y-BOCS)**	−0.058	−0.015	0.33	−0.175	0.861	−0.71–0.60	1.37
**Mindful Attention Awareness Scale (MAAS)**	0.10	0.04	0.21	0.45	0.651	−0.33–0.52	1.62
**Anxiety Sensitivity Index-3 (ASI-3)**	0.92	0.52	0.19	4.84	<0.001	0.54–1.30	2.04
**Beck Anxiety** **Inventory (BAI)**	0.52	0.21	0.27	1.91	0.059	−0.02–1.06	2.05

The model accounted for a significant proportion of variance in MSL scores (R^2^ = 0.41, adjusted R^2^ = 0.39).

**Table 5:** Hierarchical Regression Analysis Predicting Misophonia Symptoms Severity in the OCD Group.

Predictor	B	SE	β	t	*p*
**Step 1**					
**Yale-Brown Obsession and Compulsion Scale (Y-BOCS)**	−0.06	0.33	−0.02	−0.18	0.861
**Beck Anxiety** **Inventory (BAI)**	0.52	0.27	0.21	1.91	0.059
**Mindful Attention Awareness Scale (MAAS)**	0.10	0.21	0.04	0.45	0.651
**Model statistics (Step 1)**					
R^2^ = 0.218					
**Step 2**					
**Anxiety Sensitivity (ASI-3)**	0.92	0.19	0.52	4.84	<0.001
**Model statistics (Step 2)**					
R^2^ = 0.430	ΔR^2^ = 0.211	F-change = 38.18	*p* < 0.001		

**Table 6:** Mediating Role of ASI-3 in the Relationship Between Y-BOCS and MSL; Mediating Role of MAAS in the Relationship Between Y-BOCS and MSL.

(**a**)				
**Path**	**Effect**	**Std. β**	**Std. Error (Sβ)**	***p*-Value**
**a**	Y-BOCS → ASI-3	0.49	0.085	<0.001
**b**	ASI-3 → MSL	0.62	0.087	<0.001
**c (total)**	Y-BOCS → MSL	0.32	0.092	0.001
**c′ (direct)**	Y-BOCS → MSL	0.02	0.087	0.82
**a × b (indirect)**	Y-BOCS → ASI-3 → MSL	0.30	0.063	<0.001
(**b**)				
**Path**	**Effect**	**Std. β**	**Std. Error (Sβ)**	***p*-Value**
**a**	Y-BOCS → MAAS	−0.31	0.092	0.001
**b**	MAAS → MSL	−0.29	0.093	0.003
**c (total)**	Y-BOCS → MSL	0.32	0.092	0.001
**c′ (direct)**	Y-BOCS → MSL	0.23	0.093	0.015
**a × b (indirect)**	Y-BOCS → MAAS → MSL	0.09	0.044	0.009

(**a**) Y-BOCS: Yale-Brown Obsession and Compulsion Scale, MSL: Misophonia Symptom List, ASI-3: Anxiety Sensitivity Index-3; (**b**) Y-BOCS: Yale-Brown Obsession and Compulsion Scale, MAAS: Mindful Attention Awareness Scale, MSL: Misophonia Symptom List.

## Data Availability

The datasets generated and analyzed during the current study are not publicly available due to the presence of sensitive clinical and personal information but are available from the corresponding author on reasonable request, subject to approval by the local ethics committee.

## References

[1] Öztürk M.O., Uluşahin N.A. (2023). Mental Health and Disorders.

[2] Tükel R., Demet M.M. (2021). Obsessive-Compulsive and Related Disorders.

[3] Cassiello-Robbins C., Anand D., McMahon K., Guetta R., Trumbull J., Kelley L., Rosenthal M.Z. (2020). The mediating role of emotion regulation within the relationship between neuroticism and misophonia: A preliminary investigation. Front. Psychiatry.

[4] Naylor J., Caimino C., Scutt P., Hoare D.J., Baguley D.M. (2021). The prevalence and severity of misophonia in a UK undergraduate medical student population and validation of the Amsterdam misophonia scale. Psychiatr. Q..

[5] Cusack S.E., Cash T.V., Vrana S.R. (2018). An examination of the relationship between misophonia, anxiety sensitivity, and obsessive-compulsive symptoms. J. Obs.-Compuls. Relat. Disord..

[6] Reid A.M., Guzick A.G., Gernand A., Olsen B. (2016). Intensive cognitive-behavioral therapy for comorbid misophonic and obsessive-compulsive symptoms: A systematic case study. J. Obs.-Compuls. Relat. Disord..

[7] Jager I., de Koning P., Bost T., Denys D., Vulink N. (2020). Misophonia: Phenomenology, comorbidity and demographics in a large sample. PLoS ONE.

[8] Mantar A., Yemez B., Alkın T. (2011). Anxiety sensitivity and its place in psychiatric disorders. Turk. J. Psychiatry.

[9] Deacon B., Abramowitz J. (2006). Anxiety sensitivity and its dimensions across the anxiety disorders. J. Anxiety Disord..

[10] Wheaton M.G., Mahaffey B., Timpano K.R., Berman N.C., Abramowitz J.S. (2012). The relationship between anxiety sensitivity and obsessive-compulsive symptom dimensions. J. Behav. Ther. Exp. Psychiatry.

[11] Kabat-Zinn J. (2003). Mindfulness-based interventions in context: Past, present, and future. Clin. Psychol. Sci. Pract..

[12] Bishop S.R., Lau M., Shapiro S., Carlson L., Anderson N.D., Carmody J., Segal Z.V., Abbey S., Speca M., Velting D. (2004). Mindfulness: A proposed operational definition. Clin. Psychol. Sci. Pract..

[13] Hertenstein E., Rose N., Voderholzer U., Heidenreich T., Nissen C., Thiel N., Herbst N., Külz A.K. (2012). Mindfulness-based cognitive therapy in obsessive-compulsive disorder–A qualitative study on patients’ experiences. BMC Psychiatry.

[14] Külz A.K., Landmann S., Cludius B., Hottenrott B., Rose N., Heidenreich T., Hertenstein E., Voderholzer U., Moritz S. (2014). Mindfulness-based cognitive therapy in obsessive-compulsive disorder: Protocol of a randomized controlled trial. BMC Psychiatry.

[15] Ghorbani S., Ashouri A., Gharraee B., Farahani H. (2022). Effectiveness of Online Group-mindfulness and Acceptance-based Therapy and Cognitive-behavioral Therapy on Misophonia. Iran. J. Psychiatry Behav. Sci./Prog. Psychiatry Behav. Sci..

[16] Schneider R.L., Arch J.J. (2017). Case study: A novel application of mindfulness-and acceptance-based components to treat misophonia. J. Context. Behav. Sci..

[17] Schröder A., Vulink N., Denys D. (2013). Misophonia: Diagnostic criteria for a new psychiatric disorder. PLoS ONE.

[18] Edelstein M., Brang D., Rouw R., Ramachandran V.S. (2013). Misophonia: Physiological investigations and case descriptions. Front. Hum. Neurosci..

[19] Kumar S., Tansley-Hancock O., Sedley W., Winston J.S., Callaghan M.F., Allen M., Cope T.E., Gander P.E., Bamiou D.-E., Griffiths T.D. (2017). The brain basis for misophonia. Curr. Biol..

[20] Brown K.W., Ryan R.M. (2003). The benefits of being present: Mindfulness and its role in psychological well-being. J. Personal. Soc. Psychol..

[21] Özyeşil Z., Arslan C., Kesici Ş., Deniz M.E. (2011). Adaptation of the Mindful Attention Awareness Scale into Turkish. Educ. Sci..

[22] Taylor S., Zvolensky M.J., Cox B.J., Deacon B., Heimberg R.G., Ledley D.R., Abramowitz J.S., Holaway R.M., Sandin B., Stewart S.H. (2007). Robust dimensions of anxiety sensitivity: Development and initial validation of the Anxiety Sensitivity Index-3. Psychol. Assess..

[23] Goodman W.K., Price L.H., Rasmussen S.A., Mazure C., Fleischmann R.L., Hill C.L., Heninger G.R., Charney D.S. (1989). The Yale-Brown Obsessive Compulsive Scale: I. Development, use, and reliability. Arch. Gen. Psychiatry.

[24] Karamustafalıoğlu K.O., Üçışık A.M., Ulusoy M., Erkmen H. (1993). Yale-Brown Obsesyon-Kompulsiyon Derecelendirme Ölçeği’nin Geçerlilik ve Güvenilirlik Çalışması.

[25] Beck A.T., Epstein N., Brown G., Steer R.A. (1988). An inventory for measuring clinical anxiety: Psychometric properties. J. Consult. Clin. Psychol..

[26] Ulusoy M., Sahin N.H., Erkmen H. (1998). Turkish Version of the Beck Anxiety Inventory. J. Cogn. Psychother..

[27] Hayes A.F. (2018). Introduction to Mediation, Moderation, and Conditional Process Analysis: A Regression-Based Approach.

[28] Andermane N., Bauer M., Simner J., Ward J. (2023). A symptom network model of misophonia: From heightened sensory sensitivity to clinical comorbidity. J. Clin. Psychol..

[29] Blakey S.M., Abramowitz J.S., Reuman L., Leonard R.C., Riemann B.C. (2017). Anxiety sensitivity as a predictor of outcome in the treatment of obsessive-compulsive disorder. J. Behav. Ther. Exp. Psychiatry.

[30] Leeuwerik T., Cavanagh K., Strauss C. (2020). The association of trait mindfulness and self-compassion with obsessive-compulsive disorder symptoms: Results from a large survey with treatment-seeking adults. Cogn. Ther. Res..

[31] Calamari J.E., Rector N.A., Woodard J.L., Cohen R.J., Chik H.M. (2008). Anxiety sensitivity and obsessive-compulsive disorder. Assessment.

[32] Schadegg M.J., Clark H.L., Dixon L.J. (2021). Evaluating anxiety sensitivity as a moderator of misophonia and dimensions of aggression. J. Obs.-Compuls. Relat. Disord..

[33] Euser A.M., Oosterhoff M., van Balkom I. (2016). Stuck song syndrome: Musical obsessions—When to look for OCD. Br. J. Gen. Pract..

[34] Mutlu K., Tamam L., Namlı Z., Demirkol M.E., Karaytuğ M.O. (2023). Misophonia and its ‘s Relationship with Other Psychiatric Disorders. Med. Rec..

[35] Giannouli V., Yordanova J., Kolev V. (2024). Can brief listening to Mozart’s music improve visual working memory? An update on the role of cognitive and emotional factors. J. Intell..

[36] Giannouli V., Stoyanova S. (2018). Are there any links among psychopathological symptoms, musical preferences and verbal working memory in female adults?. Acta Neuropsychol..

[37] Barahmand U., Mantzikos M.S., Xiang Y., Shamsina N., Rotlevi E. (2023). Disgust and anxiety sensitivity as vulnerability factors in Misophonia. Psychiatry Behav. Sci..

[38] Yalım E., Mavi C.Ü., Hocaoğlu Ç. (2025). Investigation of Misophonia Frequency, Disgust Sensitivity, and Anger Expression Style in Patients Diagnosed with Obsessive-Compulsive Disorder. Psychiatry Behav. Sci..

[39] Thakeria K., Jain K., Sain K., Rani R., Sharma A., Sharma R. (2025). Unveiling the connection: Music preferences and mental health through machine learning analysis. Proceedings of the 2025 Fifth International Conference on Advances in Electrical, Computing, Communication and Sustainable Technologies (ICAECT).

[40] Truong T.P.A., Applewhite B., Heiderscheit A., Himmerich H. (2021). A systematic review of scientific studies and case reports on music and obsessive-compulsive disorder. Int. J. Environ. Res. Public Health.

